# Old dogs, new tricks: MR‐Linac training and credentialing of radiation oncologists, radiation therapists and medical physicists

**DOI:** 10.1002/jmrs.640

**Published:** 2022-12-11

**Authors:** Louise Hogan, Michael Jameson, David Crawford, Stacy Alvares, Conrad Loo, Maddison Picton, Zoe Moutrie, Claire Pagulayan, Ursula Jelen, Nicolle Dunkerley, Tania Twentyman, Jeremy de Leon, Vikneswary Batumalai

**Affiliations:** ^1^ GenesisCare Alexandria New South Wales Australia; ^2^ School of Clinical Medicine, Faculty of Medicine and Health UNSW Sydney Australia; ^3^ Department of Radiation Oncology South West Sydney Local Health District Warwick Farm New South Wales Australia

## Abstract

The introduction of magnetic resonance (MR) linear accelerators (MR‐Linacs) into radiotherapy departments has increased in recent years owing to its unique advantages including the ability to deliver online adaptive radiotherapy. However, most radiation oncology professionals are not accustomed to working with MR technology. The integration of an MR‐Linac into routine practice requires many considerations including MR safety, MR image acquisition and optimisation, image interpretation and adaptive radiotherapy strategies. This article provides an overview of training and credentialing requirements for radiation oncology professionals to develop competency and efficiency in delivering treatment safely on an MR‐Linac.

## Graphical abstract

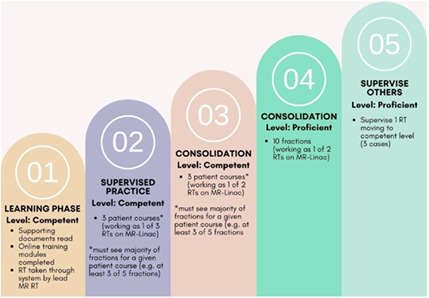

## Introduction

Over the past few years, the use of magnetic resonance (MR) in radiotherapy planning and treatment has been shown to be beneficial in overall patient care.^1^ MR technology is becoming much more prevalent in departments worldwide in the form of diagnostic MRI scanners, MR simulators and more recently MR linear accelerators (MR‐Linacs). The advancements in MR‐guided radiotherapy mean that treating clinicians can now account for anatomical changes during each treatment session through superior soft tissue visualisation of the targets and organs at risk (OARs). Subsequently, radiation therapists (RT) can re‐plan based on these day‐to‐day changes, therefore aiming to improve dosimetric accuracy and reduce toxicity by providing a fully adaptive treatment approach for patients.^2^, ^3^

Together with Elekta (Stockholm, Sweden) and Philips (Best, The Netherlands), the researchers at University Medical Centre Utrecht developed, built and clinically introduced a hybrid 1.5 T MR‐Linac.^4^, ^5^ The introduction of an MR‐Linac into radiotherapy departments has changed the paradigm where computed tomography (CT)‐based image guidance has long been the routine imaging used both for treatment planning and verification, with RTs being the frontline staff in treatment delivery.^6^ In contrast, with MR‐Linac systems, it is common practice for radiation oncologists (ROs), radiation oncology medical physicists (ROMPs) and RTs to be present at treatment delivery, consequently changing the traditional role of these professional groups (Table 1). With this shift to MR‐guided radiotherapy, it is necessary to consider MR‐Linac‐specific training and credentialing of staff that includes MR safety, MR image acquisition and optimisation for radiotherapy purposes, MR image interpretation and adaptive radiotherapy strategies.^7^, ^8^

**Table 1:** Tasks associated with online CT‐based image‐guided radiotherapy and MR‐Linac‐guided radiotherapy.

Task	CT‐based image‐guided radiotherapy			MR‐Linac‐guided radiotherapy		
	RT	ROMP	RO	RT	ROMP^*^	RO
Patient set up	√			√		
Image acquisition	√			√	√	
Image registration	√			√		
Image review/interpretation				√	√	√
Contour review/edit				√	√	√
Online plan optimisation				√		
Online plan review				√		√
Online plan QA				√	√	
Beam‐on	√			√		

* ROMP presence no longer required at the treatment console 6 months after the introduction of MR‐Linac in our department.

This article will detail the training and credentialing framework that has been developed to support competency and efficiency of our processes. We discuss how the introduction of the Elekta Unity MR‐Linac in our department has impacted ways of working and more specifically how each craft group has adapted and overcome the challenges.

## Training Framework

When looking broadly at the overall clinical team who deliver treatment on an MR‐Linac, the training framework for all three professional groups can be collectively combined into two primary categories: (1) MR safety and (2) vendor training.

### MR safety

Despite the many advantages of integrating MR into radiotherapy workflows, the dangers of MR are not commonly understood in an oncology unit. The danger stems from the fact that most of the team will have no previous experience within an MR environment and thus are unfamiliar with the impact a magnetic field can have and the associated risks. Therefore, MR safety poses the most challenging aspect of introducing an MR‐Linac to a radiotherapy department. Education is paramount to reduce risks and ensure patients and staff are both informed and protected.^9^, ^10^

We note that historically (and at the time of the initial work of this article), there was no MR‐Linac focused local accredited training offered in Australia or New Zealand in MR safety. Therefore, in accordance with the Royal Australian and New Zealand College of Radiologists (RANZCR) guidelines,^11^ we developed MR safety training to educate all staff in the department, subdivided into Level 1 and Level 2. Level 1 is compulsory for any staff member working in a department that houses an MR‐Linac, and a yearly refresher is required to maintain a competent understanding. Level 2 is required for all clinical staff working directly in the control room and treatment bunker of the MR‐Linac. The control and treatment room has restricted entry by swipe card access, which is only granted to those who have completed Level 2 training and corresponding assessments. Table 2 details the specific training requirements for various craft groups within the department. The initial focus of training was to educate the direct users (RTs, ROs and ROMPs) on the importance of MR safety, and it then became the responsibility of the nominated supervising staff from each of these craft groups to impart that knowledge on all other staff working within the department, most notably nursing and patient service officers.

**Table 2:** MR safety training requirements of specific craft groups.

	A	B	C
Level of MR safety training			
Level 1	√	√	√
Level 2	√	√	
Specific training			
Training and instructions in the use of the equipment, hazards and actions during emergency	√		
Awareness on the relevant content of the MR‐Linac instructions	√		
Awareness on the location of the MR environment and hazards	√	√	√
Understanding of the safety aspects relating to:			
The electrical safety of the equipment	√	√	
The main static magnetic field	√		
Radio‐frequency fields	√		
Gradient magnetic fields	√		
Understanding of emergency procedures arising from causes other than equipment failure	√		√
Understanding of local regulations and procedures in connection with the MR diagnostic equipment and location	√		
Understanding of the MR controlled access area, MR environment and MR projectile zone including:	√	√	
The projectile effect	√	√	
The effect of magnetic field on implants and prostheses	√	√	
The effect of magnetic field on personal effects (e.g. credit cards and watches)	√	√	
Understanding of the consequences and effects of quenching superconducting magnets	√		
Awareness of the recommendations on exposure to MR	√		
Understanding of the consequences of the correct selection, fitting and use of ear protection	√		

A – MR Radiographer/MR‐Linac Radiation therapist, Medical Physicist, Radiation Oncologist.

B – Cleaning staff.

C – All other staff who are allowed to enter the MR Controlled Access Area, but not the MR Environment.

The MR safety training included the development of a site MR safety plan, and the plan was reviewed by an external MR safety expert. While such a document is not mandated by legislation, it was valuable and enabled a single document detailing roles, responsibilities and reporting lines as recommended by RANZCR,^11^ American College of Radiology^12^ and Medicines and Healthcare Products Regulatory Agency.^13^ This detailed document provided oversight for our management team on the MR environment, to help set out clear expectations for external contractors, tradespeople, technicians and visiting staff. The MR safety plan included out of hours reporting lines to ensure clear emergency management procedures. It is noted that this robust plan was developed by ROMPs who are experienced in developing similar quality management plans in ionising radiation safety.

### Vendor training

**Figure 1:**
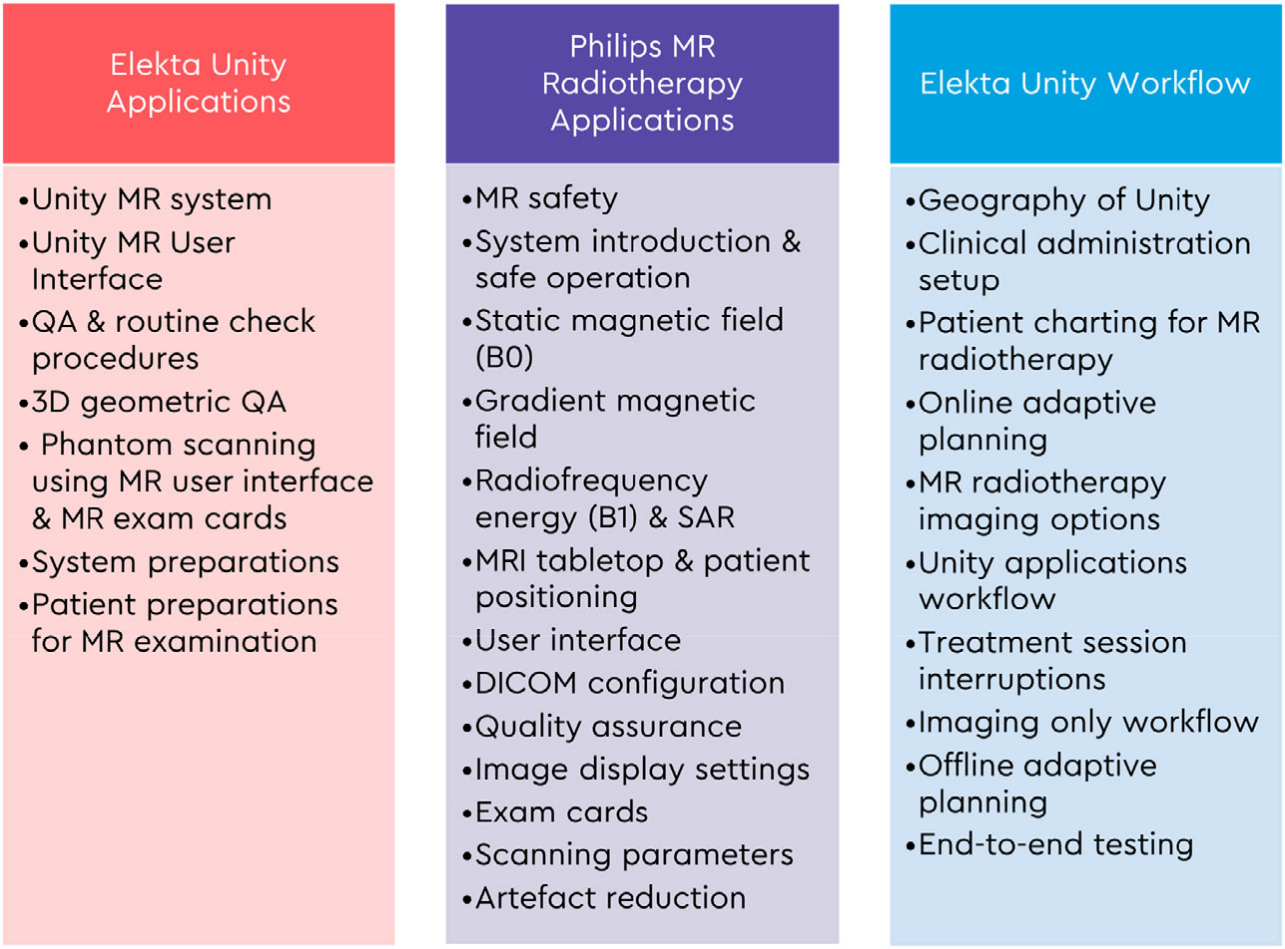
Vendor training for the treating clinical team.

The goals of the training programme provided by Elekta and Philips are to ensure that the Unity can be safely and effectively used to treat patients, and to have the adoption of Unity in the clinic as seamless as possible. Vendor training for the treating clinical team consisted of a 5‐day Unity application training and an introduction course on the use of Monaco treatment planning system, 5 days of Philips MR radiotherapy application training and further 5 days of Elekta Unity application workflow and end‐to‐end training (Figure. 1). This training also included characteristics unique to the Unity including physical limitations of the machine (fixed isocentre, step and shoot beams, avoiding beam entry through the cryostat pipe), electron streaming and return effects and differences in planning techniques. An additional 10 days of training and validation specific for ROMPs was provided by Elekta that included dosimetry calibration, Unity MLC, cryostat and beam characterisation, patient‐specific quality assurance (QA), image orientation and beam model validation.

In the initial few months after the introduction of the MR‐Linac in our department, only staff who had completed the MR safety and vendor training were involved in patient treatment. This period was considered as on‐the‐job training for the clinical team. Over time, more staff were onboarded requiring the initiation and application of the MR‐Linac credentialing programme.

## MR‐Linac Credentialing (Assessing and Approving)

A credentialing framework specific for each craft group was created to support staff to develop skills and become confident and competent in MR‐Linac safety, principles and procedures. This framework includes assessments for any new staff rotating onto the MR‐Linac. Staff must demonstrate competence in the clinical and knowledge‐based activities identified in the framework. Demonstration of clinical competence will confirm that the individual's trainer has observed the staff performing the procedure independently, consistently and effectively. For ROs, the focus is on developing proficiency in MR‐based contouring, efficiency with plan reviews in Monaco and becoming credentialed for the role of the treating RO during online adaptive treatments. This was further broken down to credentialing for different clinical indications such as stereotactic prostate, oligometastatic nodes in the abdomen and pelvis, liver and pancreas. For ROMPs, proficiency is required in MR‐Linac QA, MR‐Linac dosimetry, patient‐specific QA and on‐treatment support. RTs are required to upskill and be credentialed in all aspects of MR and CT simulation, planning and treatment of patients on the MR‐Linac, focusing on the two different online workflows, adapt‐to‐position (ATP) and adapt‐to‐shape (ATS).^14^

**Figure 2:**
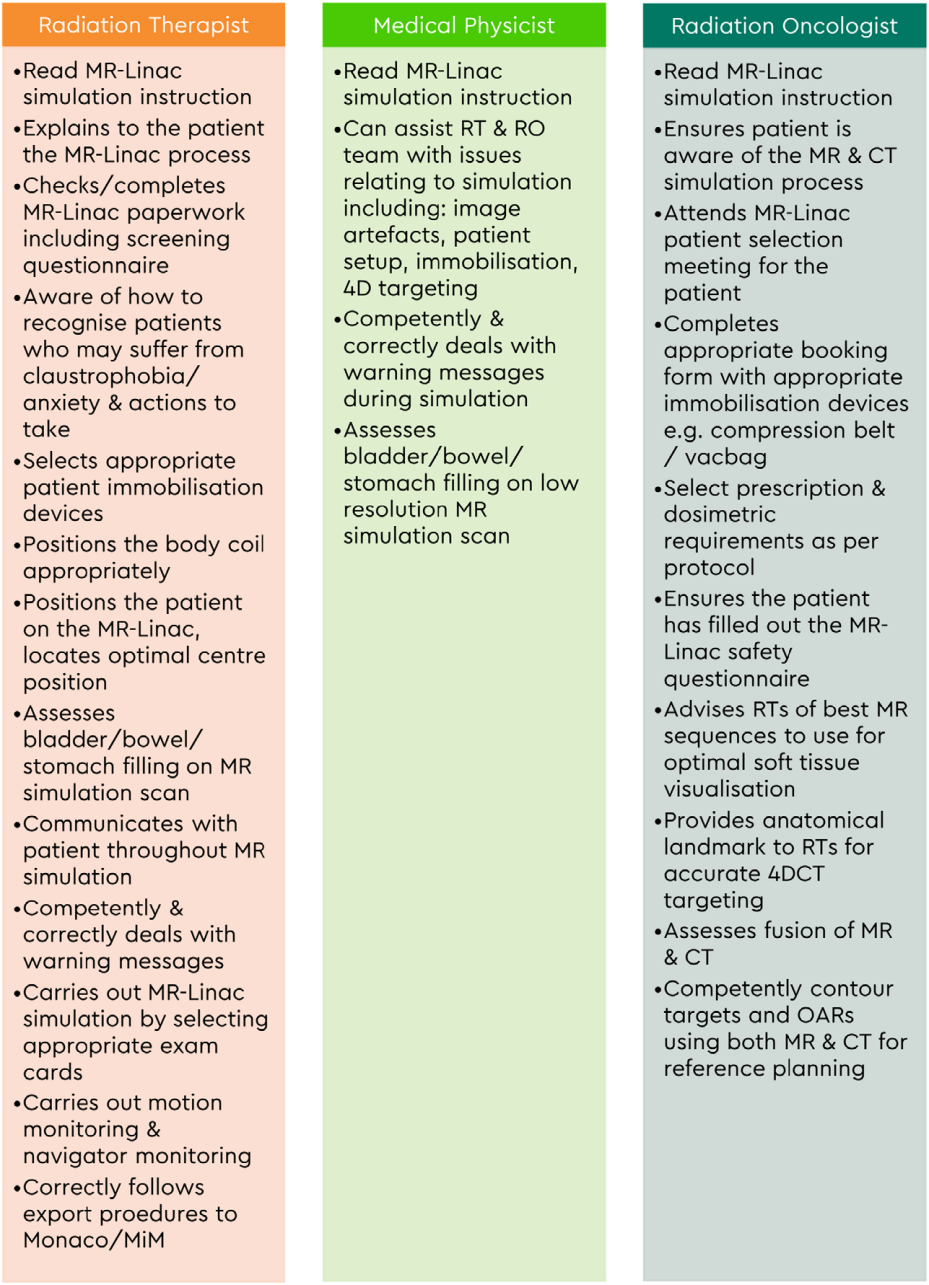
MR‐Linac simulation knowledge and skills indicators.

**Figure 3:**
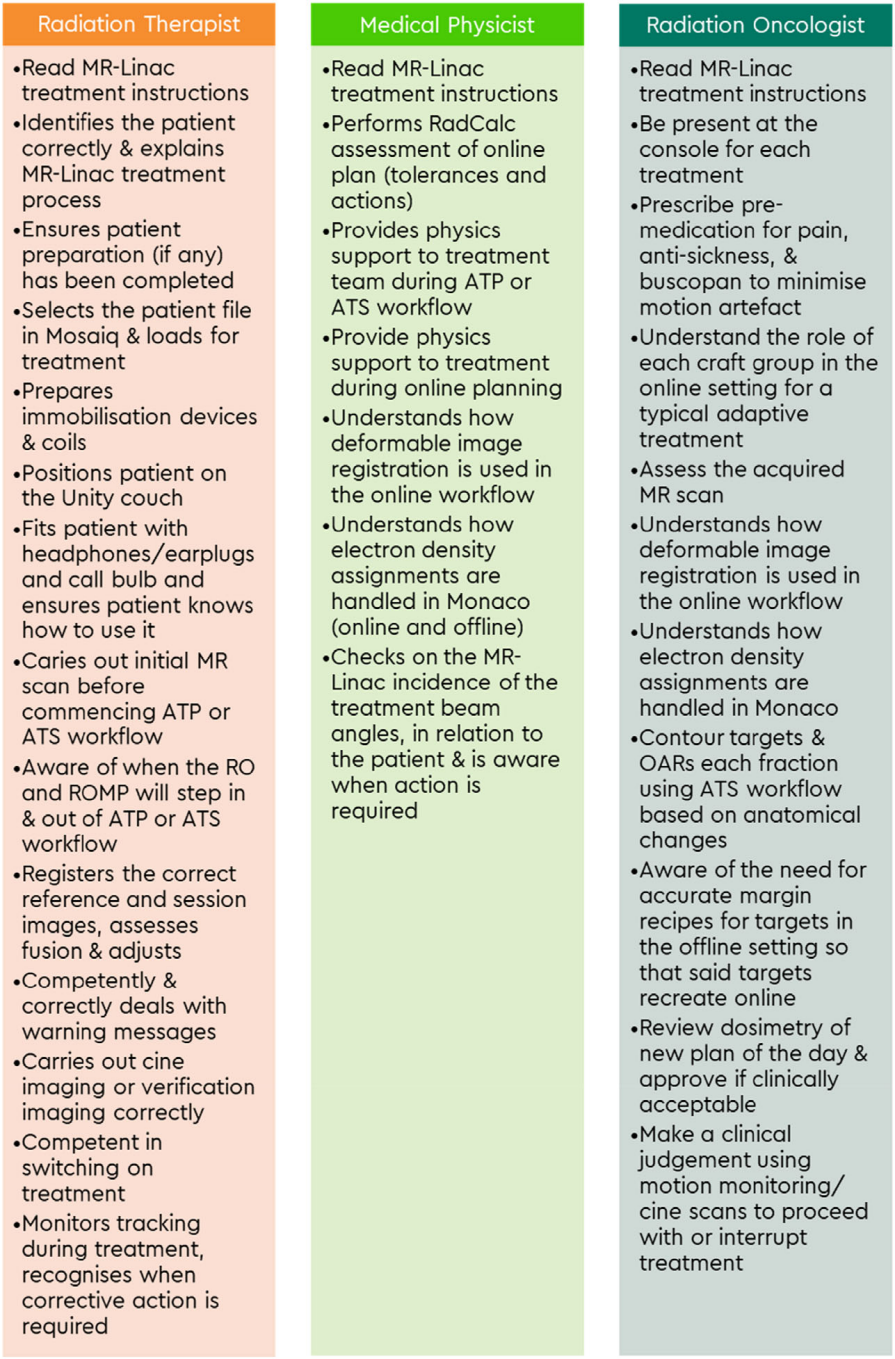
MR‐Linac treatment knowledge and skills indicators.

General credentialing items for all three professional groups include patient care (Table 3) and safety and knowledge of the MR‐Linac system (Table 4). The clinical team are also required to log a minimum of 20–40 h in the MR‐Linac environment. Figure 2 highlights the knowledge and skills to be assessed for MR‐Linac simulation while Figure 3 shows the MR‐Linac treatment knowledge indicators.

**Table 3:** Assessment of patient care and safety.

Knowledge Indicators
Read all MR‐Linac‐related safety documents
Identify the MR‐Linac safety zones and how they can affect patient and staff safety and MR‐Linac access
Explain the terms MR Safe, MR Conditional and MR Unsafe in relation to patient and staff safety and the associated labelling of equipment
Explain the term projectile hazard and the associated risk to patient safety
Explain the term bioeffects in relation to patient safety
Explain the risk of peripheral nerve stimulation to patients undergoing MR‐Linac procedures
Explain the risk of acoustic noise and how to minimise the risk in the MR‐Linac environment
Explain the risk of patient ‘loops’ and how to minimise the risk
Explain the term SAR and the influence of patient weight on it
Explain the risks to patient safety during a magnet quench, when a quench should take place and what steps to take if such an event should occur
Explain the importance of the MRL Patient Safety Questionnaire
Explain the steps to take if a patient has a conditional implant or device
Explain the correct action to take if a patient emergency was to take place inside the MR‐Linac unit
Explain the terms MR Safety Expert, MR Designated Responsible Person, MR Safety Officers and MR safety committee
Explain staff screening requirements and MR‐Linac access rights

**Table 4:** Knowledge of the MR‐Linac system.

Knowledge Indicators
Identify the location of emergency stop buttons and quench buttons
Identify the location of five gauss line and understands the importance of it
Identify the major hardware components of the MR‐Linac including magnet, gantry, gradient coils, radiofrequency transmit coils, multi‐leaf collimators etc.
Identify the MR imaging sequence used on the MR‐Linac system
Competent in the use of the patient immobilisation devices system
Competent in the use of the body coil and understands why it is used
Correct use of the patient safety aids – earphones and call bulb
Correct use of the patient safety devices – intercom and video cameras
Competent in the use of the RT operator console computers
Aware of faults that may appear on the MR‐Linac system
Explain the MR‐Linac fault recording and reporting process

**Figure 4:**
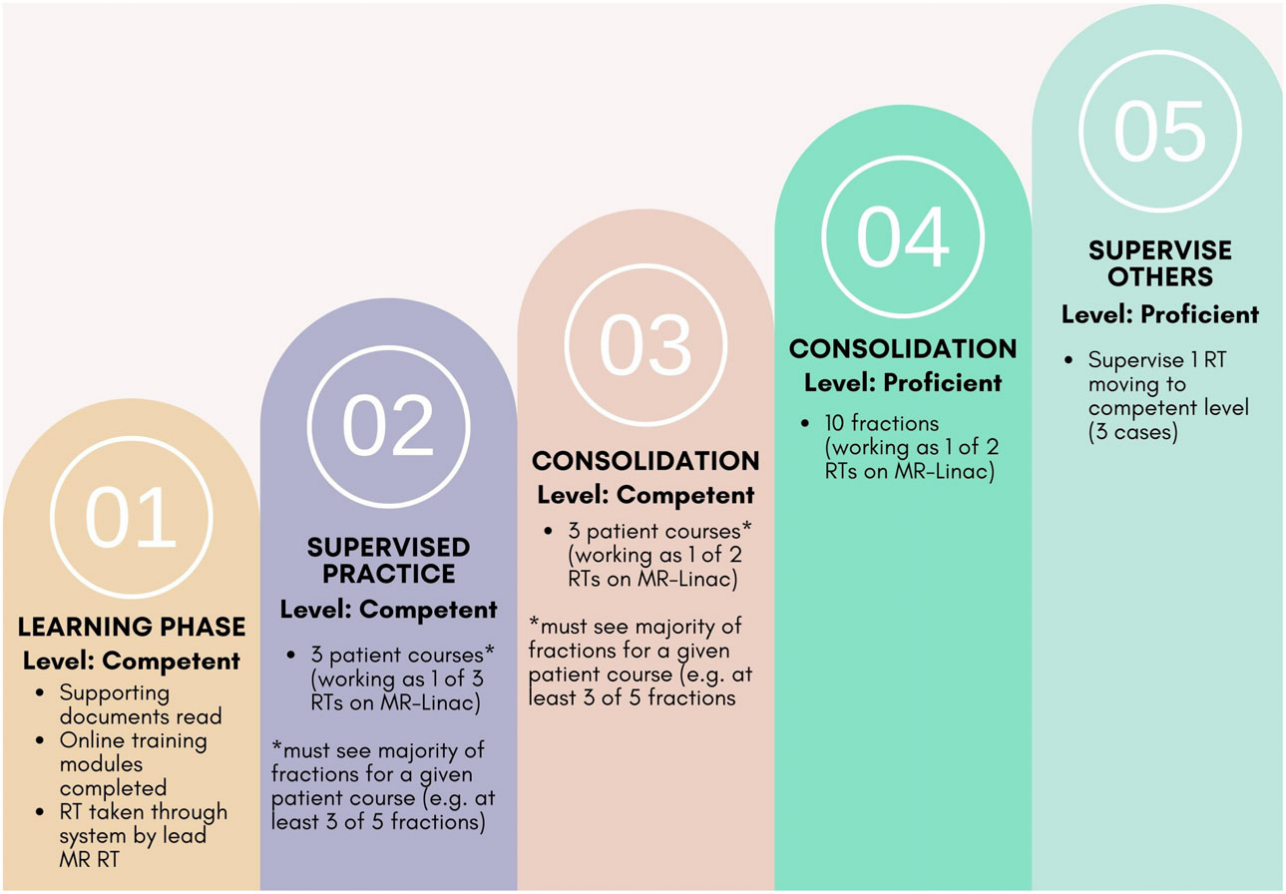
Radiation therapist treatment competency assessment.

The competency assessments for RTs comprise five different steps which demonstrate competency progression from ‘competent’ to ‘proficient’ through the learning phase, supervised practice, consolidation and supervision of others (Fig. 4). These assessments are first completed for pelvis treatment and are required to be repeated for each additional clinical indication (e.g. abdomen). For ROMPs, the competency assessments are divided into two frameworks: QA (patient QA, dosimetry and routine QA) and treatment support. To be signed off as competent for each framework, ROMPs are required to read supporting documents, complete online training modules and provide one case demonstration (perform and analyse the machine and patient‐specific QA to verify the safe and consistent operation of the MR‐Linac). Credentialing of ROs for treatment on the MR‐Linac is based on their ability to demonstrate: (1) observational assessment (observe two live MR‐Linac treatments), (2) peer review (attending the MR‐Linac patient selection meeting and undertake the planning of at least five cases and peer reviewed prior to treatment) and (3) supervised practice (perform five treatments on the MR‐Linac under the supervision of a credentialed RO). Credentialing logs are updated on a yearly basis to ensure continual competency is maintained for all craft groups.

## Discussion

The robust and extensive MR‐Linac training and credentialing programme developed and implemented in our centre has served to adequately educate and protect all patients and staff. This group includes ROs, RTs, ROMPs, nursing, patient service officers, cleaners and those from administration and research teams. In addition to basic MR safety training, the vendor training from both Philips and Elekta provided the clinical team with a heightened awareness and understanding of the concepts and practice of MR, along with extensive knowledge of the planning and treatment workflows associated with the Elekta Unity MR‐Linac. As our MR‐Linac programme progressed through increased patient throughput as well as commissioning of new sites, the credentialing framework outlined for each craft group has served to help upskill new staff members in a systematic and timely manner while ensuring excellent patient care. The initial competency profile (for pelvis) served as a baseline for the introduction of additional treatment sites, including liver, pancreas, bladder etc. Feedback to date from the staff who received training and the wider treatment team that have since collaboratively worked with these team members has been positive.

The role of ROs, RTs and ROMPs has evolved and expanded with the addition of the MR‐Linac, in an exciting era of adaptive radiotherapy. In many ways, this has positively influenced engagement as each craft group rises to the challenge of more responsibility and increased hands‐on involvement during a patient's treatment session.^15^ However, it has proven to be very resource intensive and can put stress and burden on other aspects of the treatment centre.^2^ In the first 6 months of clinical use in our centre, the MR‐Linac required an RO, ROMP and 2 RTs to be present at the console for every treatment session, and this meant their availability to undertake other tasks and roles was limited. As patient numbers increased, training and credentialing new staff became a challenge. Centres globally are moving towards RT‐led treatment workflows, making the traditional RT role more specialised.^16^, ^17^, ^18^, ^19^, ^20^

Another challenge faced in terms of MR safety was the different approach required for the emergency procedures within an MR environment. Part of the training materials included educating the team, in particular the nursing team, to ensure awareness of the different emergency code response procedure that should be followed in the presence of a magnetic field. In line with RANZCR recommendations,^11^ in the event of a patient emergency, the patient must be evacuated from the MR‐Linac treatment room, by MR trained personnel only, into a safe environment outside the control room and only then can the nursing team perform resuscitation. This is to prevent non‐MR safe equipment being brought into the room which could interact with the magnetic field, creating a projectile, thereby posing significant danger to both staff and patients in the bunker. MR safety and education was the number one priority of staff training in our department, particularly those with limited experience in an MR environment or familiarity with such technology. A significant emphasis was placed on highlighting the risks and associated dangers of a 1.5 T magnet. Safety presentations were conducted weekly in our centre before the magnet was ramped up and annual refreshers are compulsory.

From an RO perspective in terms of MR safety, it is important that patients are appropriately assessed for suitability for treatment on the MR‐Linac. This is done by getting the patient to complete an MR screening form at initial consultation, allowing adequate time for the RTs to review and investigate responses in advance of patient simulations. This, combined with daily MR screening, ensures any important patient changes between appointments are picked up prior to proceeding with any MR. Daily MR screening was found to be valuable as patients from time to time had medical interventions that resulted in MR conditional inorganic materials being implanted between simulation and treatment or even between fractions.

Seeing MR images day to day for both contouring and planning is something a radiotherapy team is typically not accustomed to. Most RTs have a very basic level of MR comprehension through fusions of diagnostic MR images with the simulation CT at the planning stage. Within the standard treatment pathway, cone‐beam CT to CT registrations are routinely used, limiting RT's experience with MR image interpretation. RT's lack of experience in assessing and decision‐making with MR imaging presented significant challenges. The development and implementation of robust training standards helped to overcome the aspects of this challenge, particularly through education in the interpretation of MR images. Both the imaging modality and workflow of MR‐guided radiotherapy are unique, requiring a bespoke competency profile. While the scan protocols permitted on the MR‐Linac are limited by the manufacturer, we found training and the development of quick guides on the common issues such as signal loss or artefact generation on MR to be beneficial.

While RO contouring on diagnostic or simulation MR scans during the radiotherapy planning process is not new, the ROs must now adjust to contouring online on treatment MR scans while the patient is on the bed, and this involves some level of upskilling. Since contouring is such a significant part of the clinical online adaptive workflow, an effective credentialing framework is one of the most important and useful additions to the clinical roll out. Furthermore, based on the rostering model and availability in our practice, different ROs are rostered to the MR‐Linac day to day, meaning they contour each other's patients too. A weekly peer review is conducted by the MR‐Linac team, with a representative from each craft group in attendance, and this allows the team to discuss new patients, assess the quality of the MR acquired at simulation, review contours and analyse the treatment plan and dosimetric results. This is typically a 30‐min meeting that has proven to be an invaluable forum for discussing challenging cases or cases that are an exception to the usual standard of care. It ensures clinicians and the treating team are informed and aware of what to expect in the online setting for each individual patient and ensures established treatment protocols are being followed. It can also be beneficial to have a radiologist on board to collaborate with the RO and assist in contouring the less defined lesions and provide a clear guide for contouring online each day. This is something that our centre has benefited from and has proven useful, particularly for patients receiving treatment to the liver.

## Conclusion

The MR‐Linac training and credentialing framework developed in our centre provided a structured approach to include the components of education and assessment for radiotherapy professionals. The standardised education and assessment package has facilitated a unified and efficient approach to MR‐Linac training. The framework served as a model for standardising meaningful continuing staff education and assessment in a radiotherapy clinic.

## Conflict of Interest

The authors declare no conflict of interest.
